# Development and Validation of a Three-Gene-Based Prognostic Model for Predicting the Overall Survival of Head and Neck Squamous Cell Carcinoma Through Bioinformatics Analysis

**DOI:** 10.3389/fgene.2021.721199

**Published:** 2022-01-03

**Authors:** Yiyuan Han, Xiaolin Cao, Xuemei Wang, Qing He

**Affiliations:** ^1^ The Fourth Clinical Medical College of Zhejiang Chinese Medical University, Hangzhou, China; ^2^ Department of Otorhinolaryngology and Head Neck Surgery, Affiliated Hangzhou First People’s Hospital, Zhejiang University School of Medicine, Hangzhou, China

**Keywords:** head and neck squamous cell carcinoma, prognostic model, survival analysis, cox regression, lasso algorithm

## Abstract

Head and neck squamous cell carcinoma (HNSCC) is one of the most common cancer worldwide and seriously threats public health safety. Despite the improvement of diagnostic and treatment methods, the overall survival for advanced patients has not improved yet. This study aimed to sort out prognosis-related molecular biomarkers for HNSCC and establish a prognostic model to stratify the risk hazards and predicate the prognosis for these patients, providing a theoretical basis for the formulation of individual treatment plans. We firstly identified differentially expressed genes (DEGs) between HNSCC tissues and normal tissues *via* joint analysis based on GEO databases. Then a total of 11 hub genes were selected for single-gene prognostic analysis to identify the prognostic genes. Later, the clinical information and transcription information of HNSCC were downloaded from the TCGA database. With the application of least absolute shrinkage and selection operator (LASSO) algorithm analyses for the prognostic genes on the TCGA cohort, a prognostic model consisting of three genes (COL4A1, PLAU and ITGA5) was successfully established and the survival analyses showed that the prognostic model possessed a robust performance in the overall survival prediction. Afterward, the univariate and multivariate regression analysis indicated that the prognostic model could be an independent prognostic factor. Finally, the predicative efficiency of this model was well confirmed in an independent external HNSCC cohort.

## Introduction

HNSCC is the sixth most common cancer worldwide with increasing incidence and unpredictable prognosis (Ferlay et al., 2019). The initiation and development of HNSCC involve complex molecular changes, and environment exposure, viral infection and unhealthy lifestyle are the common risk factors (Johnson et al., 2020). Despite advances in treatment and diagnostic methods, the overall survival for advanced cancers remains dismal (Price and Cohen, 2012). Therefore, elucidating the molecular mechanisms related to the tumorigenesis of HNSCC is of great importance to predicate and improve the prognosis of patients. Although The TNM staging system is one of the most extensively used indicator for monitoring HNSCC progression, it is very limited to further distinguish the clinical outcome of patients at the same TNM stage (Huang et al., 2019; Zhao and Cui, 2019). Therefore, it is critical to find a novel and reliable prognostic modules model for successful clinical managements and personalized medicine.

In our study, we established a prognostic model based on the differential expressed genes in HNSCC to stratify the risk hazards for these patients. And this three-gene prognostic model was further successfully validated as an independent prognostic factor. The robust efficiency for overall survival prediction was well validated in an independent external cohort. The prognostic model could help clinicians to estimate the prognosis of HNSCC patients and select an optimal treatment.

## Methods and Materials

### Data Sources

Three transcription profile datasets of HNSCC (GSE107591 (Verduci et al., 2017),GSE29330 (Demokan et al., 2013),GSE58911 (Lobert et al., 2014) were downloaded from GEO databases (https://www.ncbi.nlm.nih.gov/geo/) according to the following criteria:

1) Number of tumor samples ≥10; 2) The datasets contain HNSCC samples of different primary sites including the oral cavity, pharynx, larynx etc.; 3) The patients in the datasets have not received radiotherapy or chemotherapy before the surgery; 4) Gene-knockout samples should not be contained in the datasets.

Besides, the RNA-seq transcriptome data and corresponding clinical information of HNSCC samples as well as the RNA-seq transcriptome data of normal control samples were downloaded from TCGA database (including 501 HNSCC samples and 44 normal samples) (https://portal.gdc.cancer.gov/). And GSE65858 (Wichmann et al., 2015) which contains a total of 270 tumor samples was selected to verify the robustness of the prognostic model based on the following criteria:

1) Number of tumor samples ≥200; 2) The datasets contain HNSCC samples arising from different primary sites including the oral cavity, pharynx, larynx, etc.; 3) The patients in the datasets have not received radiotherapy or chemotherapy; 4) Specific gene-knockout samples should not be contained in the datasets; 5) The clinical data is complete (including gender, age, survival status, survival time, TNM staging, etc.).

### Identification and Functional Enrichment Analyses of DEGs

The GEO2R online analysis tool (https://www.ncbi.nlm.nih.gov/geo/geo2r/) was applied to identify DEGs between HNSCC and normal samples of the 3 microarray datasets downloaded from GEO databases. The adjusted *p*-value<0.05 and |log_2_ fold change (FC)|>1 were chosen as the cut-off threshold. And the Venn diagram tool (http://bioinformatics.psb.ugent.be/webtools/Venn/) was used to identify the intersecting part. GO annotation and KEGG pathway enrichment analyses were performed in the DAVID (https://david.ncifcrf.gov/) database and the adjusted *p*-value<0.05 was considered statically significant.

### PPI Network Construction and Prognostic Genes Screening

The PPI network of DEGs was constructed by STRING database (https://www.string-db.org/) and Cytoscape software (Version 3.7.1). And interactions that met the cutoff criteria, combined score>0.4, were considered statistically significant. The cytoHubba, a plugin in Cytoscape, was applied to circulate the degree value of each gene. And genes with degree>10 were regarded as hub genes. Then the survival analyses of hub genes were performed using Kaplan-Meier survival curve in the GEPIA (http://gepia.cancer-pku.cn/) databases. The hub genes with *p* < 0.05 in Kapan-Meier curve were identified as the prognostic genes.

### Differential Expression and Correlation Analysis of the Prognostic Genes in TCGA Database

Perl software (version 10.0.0) and ensemble databases were used to process original data downloaded from TCGA databases. And the expression values of prognostic genes in each sample was extracted from each sample and was visualized with a heat map. Then we circulated the median values of tumor samples and normal samples separately, performing the wilcoxon rank sum test on them to circulate the *p* value to further verified the differential expression of these prognostic genes. In addition, the pearson correlation analysis was performed to reveal the association among different prognostic genes and the results were visualized by the corrplot package in R software.

### Establishment and Validation of the Prognostic Model

Clinical data downloaded from TCGA databases were further processed, and those samples with incomplete clinical information were eliminated. A total of 395 clinical samples were identified finally (Table 1). The prognostic genes were selected for the establishment of a prognostic model with the LASSO algorithm, which was designed for variable selection and shrinkage. The standardized expression matrix of candidate prognostic DEGs was set as the independent variable in the regression, and the response variables were OS and patient status in the TCGA cohort. Three prognostic genes and their coefficients were screened with the minimum criteria, choosing the best parameter λ associated with the smallest 10-fold cross-validation error. The formula was established as follows:

$$\mathrm{Risks core}=\sum_{i=0}^{n}Coef\left(i\right)\times x\left(i\right)$$

**TABLE 1:** The clinical information of the HNSCC patients in TCGA datasets and GSE65858.

Clinicopathological features	TCGA datasets (n = 395)	GSE65858 (n = 270)
State of survival		
Dead	144	94
Alive	251	176
Age		
＞65	133	184
≤65	262	86
Gender		
Male	289	223
Female	106	47
Stage		
1–2	75	55
3–4	320	215
T		
1–2	140	115
3–4	255	155
N		
0–1	168	126
2–3	227	144
M		
0	386	263
1	9	7

The risk scores of each HNSCC patient was calculated according to the formula, and all samples were stratified into the high-risk group and the low-risk group based on the median value of the risk scores. The Kaplan-Meier survival curve and the Log-rank test were used to compare the overall survival rate of each group. Then the Receiver operating characteristic (ROC) curve was applied to evaluate the prediction accuracy of this model based on the survivalROC package. In addition, the independent prognostic value analysis was performed by univariate and multivariate Cox regression. Finally, survival and independent prognostic analyses were used in the external verification dataset (Table 1) to verify the predictive performance of the model.

## Results

### Identification and Functional Enrichment Analyses of DEGs

**FIGURE 1:**
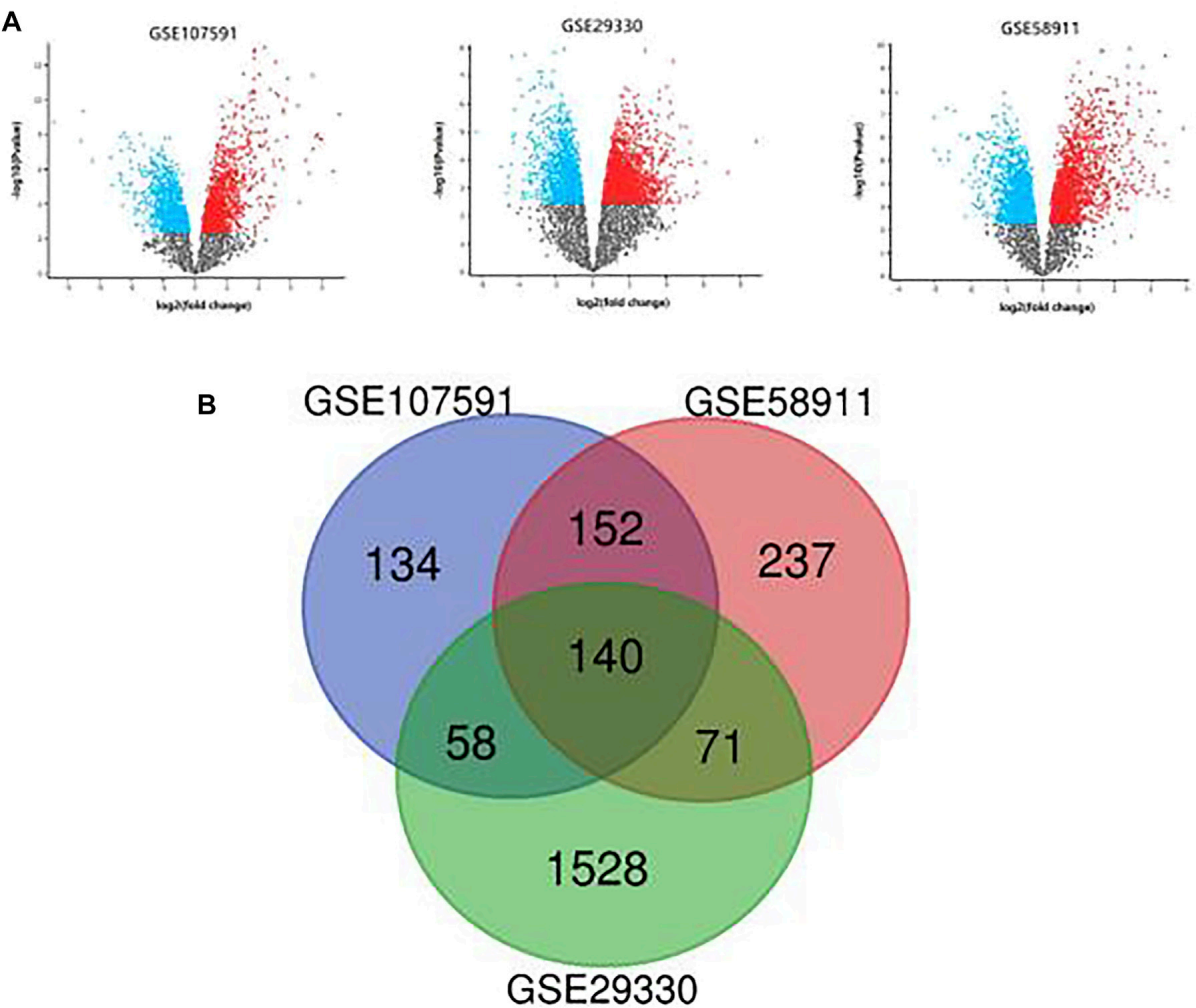
**(A)**: The differential expression results of 3 GEO datasets. **(B)**: Venn diagram of DEGs common to 3 GEO datasets.

Three transcription profile datasets of HNSCC were downloaded from GEO databases, including GSE107591, GSE29330 and GSE58911. And the expression results were present in the volcano plot (Figure 1A). Based on the criteria of adjusted *p*-value<0.05 and |log_2_ fold change (FC)|>1, there were 282 upregulated genes and 202 downregulated genes identified in GSE107591 dataset, while 1,045 upregulated genes and 752 downregulated genes were identified from GSE29330 dataset. Additionally, a total of 600 deregulated genes were identified from GSE58911 dataset, including 427 upregulated genes and 173 downregulated genes. The intersecting part of 3 databases was visualized in Venn diagram (Figure 1B), consisting of 88 upregulated DEGs and 52 downregulated DEGs.

**FIGURE 2:**
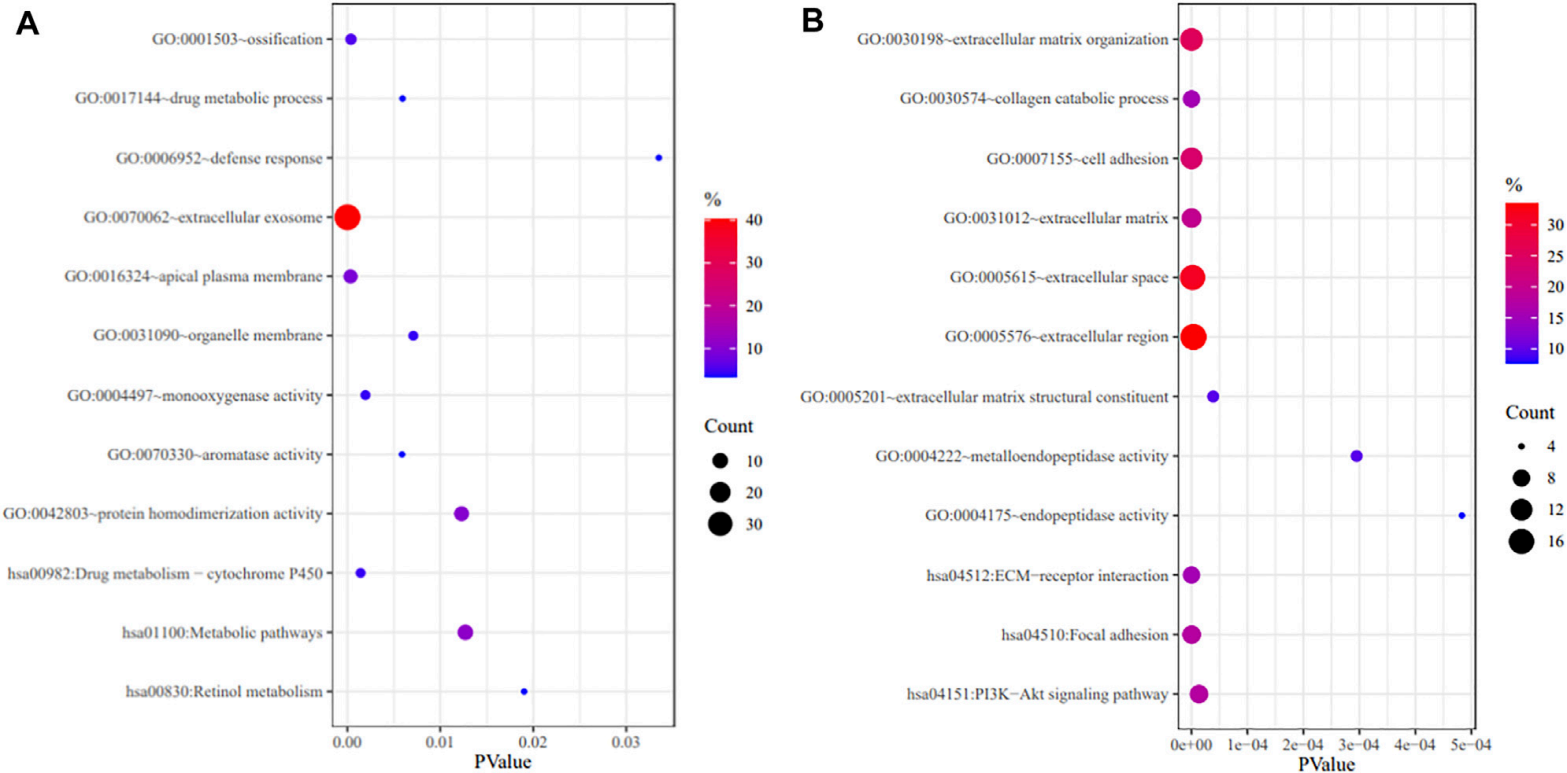
GO annotation and KEGG pathway enrichment analysis of upregulated DEGs **(A)** and downregulated DEGs **(B)**.

GO annotation and KEGG pathway enrichment analyses were performed through the DAVID databases (Figure 2). GO annotation analysis showed that the upregulated DEGs were mainly enriched in ossification, drug metabolic process, defense response, monooxygenase activity etc. As for the downregulated DEGs, the changes of GO terms were mainly enriched in extracellular matrix organization, collagen catabolic process, cell adhesion etc. The KEGG pathway enrichment analyses demonstrated that the upregulated DEGs were mainly enriched in drug metabolism, metabolic pathway, and retinol metabolism while downregulated DEGs were mainly enriched in ECM-receptor interaction, focal adhesion and PI3K-Akt signaling pathway.

### PPI Network Construction and Prognostic Genes Screening

**FIGURE 3:**
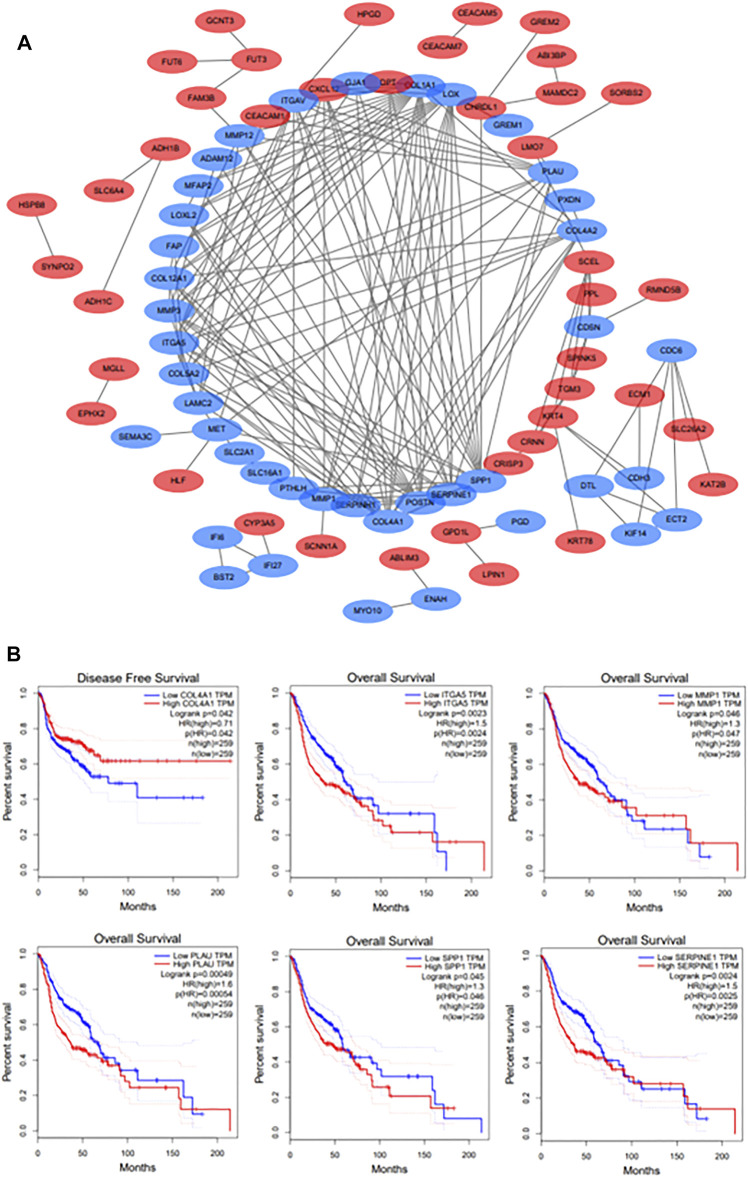
**(A)**: The PPI network of DEGs. Upregulated DEGs are marked in red; downregulated DEGs are marked in blue. **(B)**: The survival analyses of hub genes. *p* < 0.05 was considered statistically significant.

The PPI network of DEGs was constructed though Cytoscape software (Figure 3A). And a total of 11 hub genes were identified based on the criteria of degree value > 10, consisting of COL1A1, POSTN, SPP1, LOX, COL4A1, SERPINE1, ITGAV, MMP1, PLAU, ITGA5 and MMP3. The Kaplan-Meier survival curve indicated that HNSCC patients with ITGA5, MMP1, PLAU, SERPINE1, and SPP1 alterations had a worse overall survival while those with higher COL4A1 expression had a better disease-free survival (Figure 3B). These genes were considered as the prognostic genes for the construction of the prognostic model.

### Differential Expression of 6 Prognostic Genes in TCGA Datasets and Correlation Analysis

**FIGURE 4:**
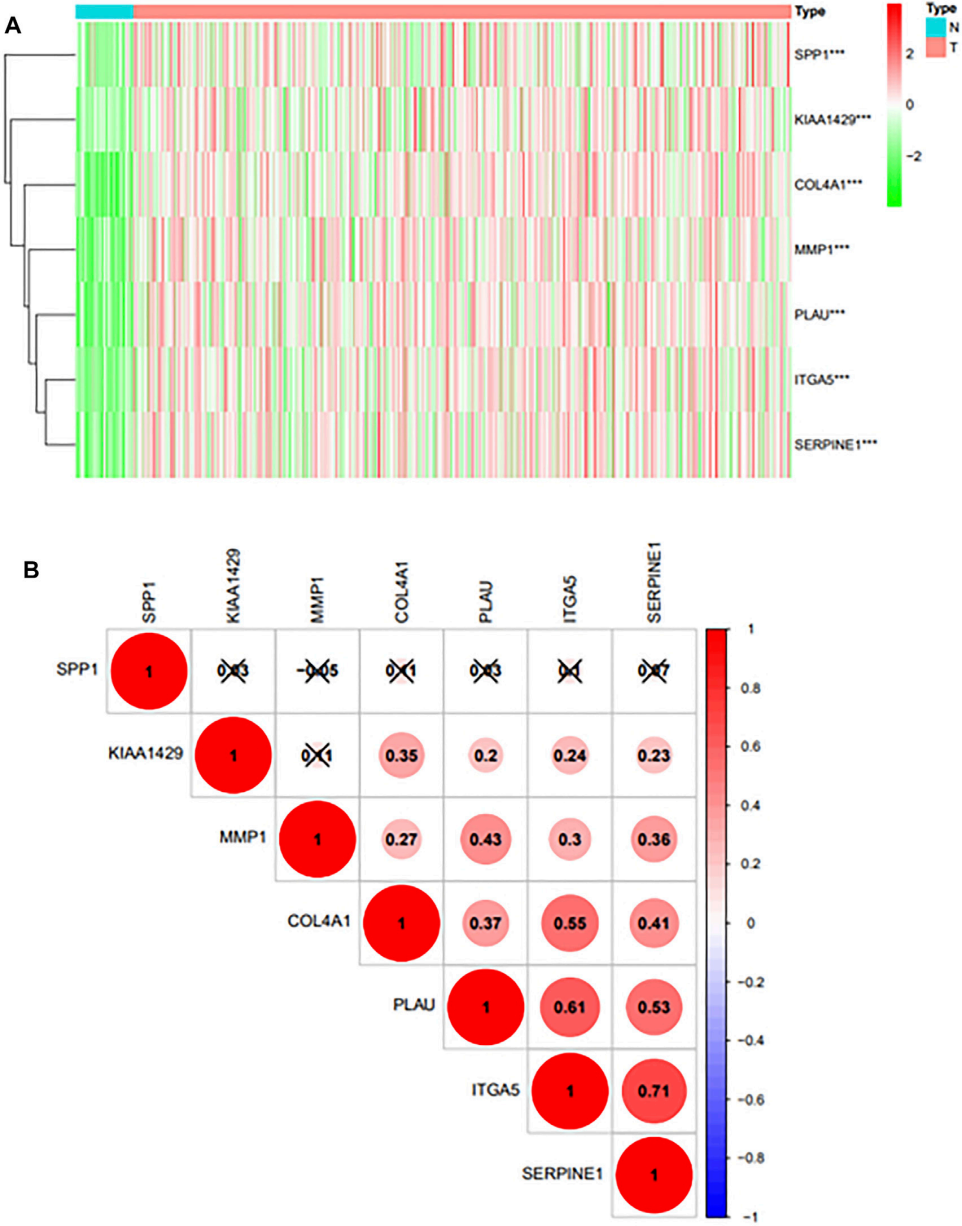
**(A)**: Differential expression of prognostic genes in TCGA databases.“***” represents “*p* < 0.05”; “**” represents “*p* < 0.01”; “***” represents “*p* < 0.001”. **(B)**: The Pearson correlation analysis results of 6 prognostic genes.

Heatmap was generated to visualize the expression pattern of 6prognostic gene between HNSCC cases and normal controls. Green or red color in the plots represented relatively low or high expression, respectively (Figure 4A). As is shown in the heatmap, all prognostic genes were upregulated in HNSCC tissues, and the *p*-value of each gene was less than 0.001. And the pearson correlation analysis revealed that ITGA5 was most correlated with SERPINE (r = 0.71) among all the interactions of the prognostic genes (Figure 4B).

### Establishment of the Prognostic Model

**FIGURE 5:**
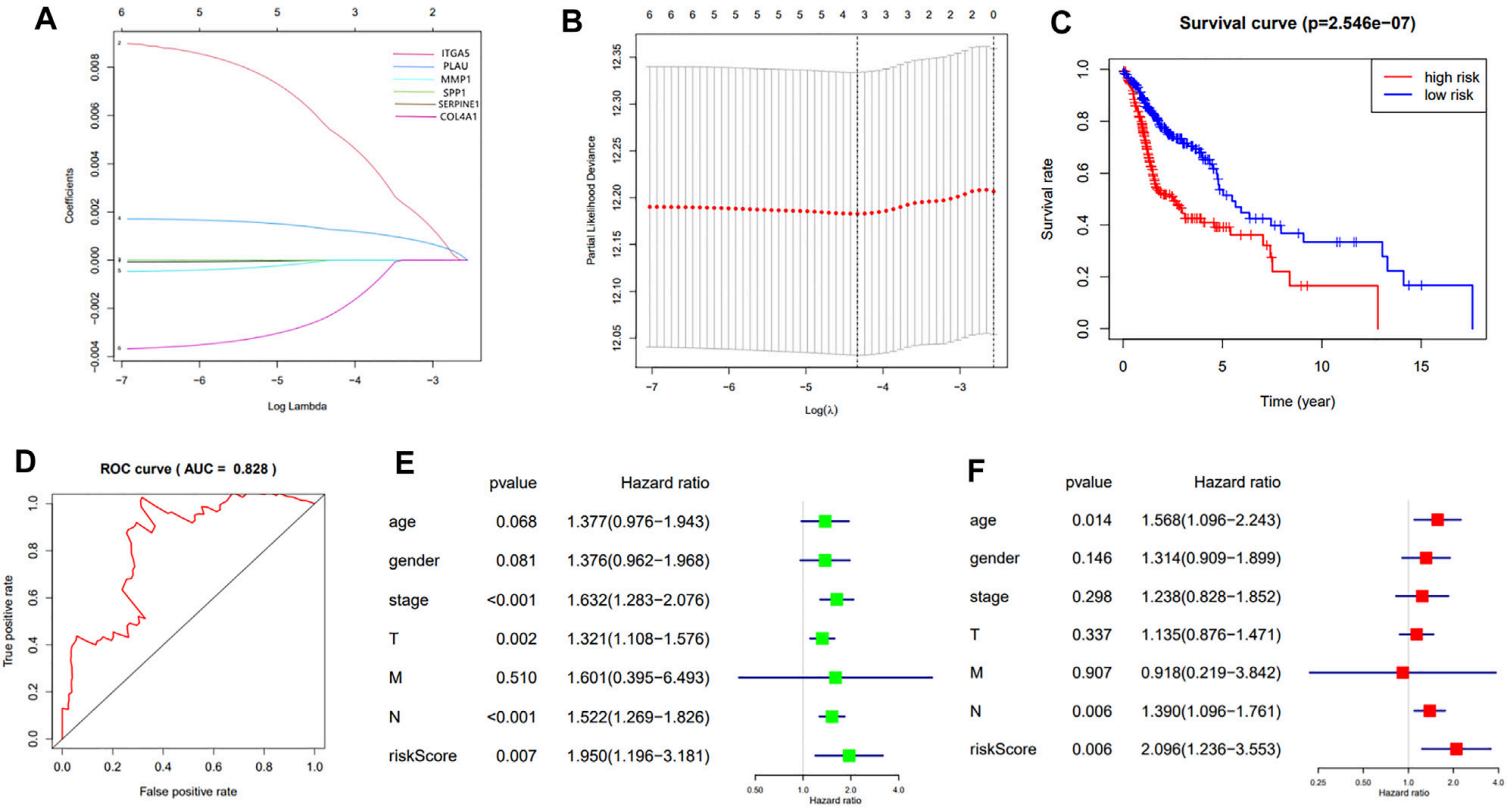
**(A)**: LASSO coefficient profiles of the 7 prognosis-related genes. A coefficient profile plot was generated against the log (λ) sequence; **(B)**: Selection of the optimal parameter (λ) in the LASSO model. The results of LASSO algorithm and cross-validation; **(C)**: The Kaplan-Meier survival curve of HNSCC patients in the TCGA cohort; **(D)**: The ROC curve and AUC value of the prognostic model; **(E,F)**: The results of the univariate and multivariate Cox regression analyses.

These prognostic genes were chosen to construct the prognostic model based on the LASSO algorithm and cross-validation (Figures 5A,B). The standardized expression matrix of candidate prognostic DEGs was set as the independent variable in the regression, and the response variables were OS and patient status in the TCGA cohort. Three genes, including COL4A1, PLAU and ITGA5, and their coefficients were identified with the minimum criteria, choosing the best parameter λ associated with the smallest 10-fold cross-validation error. The risk score of each HNSCC patient was calculated with the following formula: risk score = [ COL4A1 × (−0.00225) + PLAU × (0.00130) + ITGA5 × (0.00517) ].

According to the median of risk value the HNSCC patients in TCGA cohort were grouped into the high-risk group and low-risk group. The survival analysis showed that the patients in the high-risk group had a significantly worse overall survival than those in low-risk group (*p* = 2.546e-07) (Figure 5C). The predication efficiency of this model was well verified in the ROC curve with the AUC value equal to 0.828 (Figure 5D).

Univariate and multivariate Cox regression analyses were performed to evaluate the independent predictive value of this prognostic model. As is shown in the univariate analysis, the stage (*p* ＜0.001, HR = 1.632, 95% CI = 1.283–2.076), T-staging (*p* = 0.002, HR = 1.321, 95% CI = 1.108–1.576), N-staging (*p* < 0.001, HR = 1.522, 95% CI = 1.269–1.826) and risk score (*p* < 0.007, HR = 1.950, 95% CI = 1.196–3.181) were significantly associated with the OS (Figure 5E).The multivariate analysis showed that the N-staging (*p* = 0.006, HR = 1.390, 95% CI = 1.096–1.761) and risk score (*p* = 0.006, HR = 2.096, 95% CI = 1.236–3.553) were independent prognostic indicators (Figure 5F).

### Validation of the Prognostic Model

**FIGURE 6:**
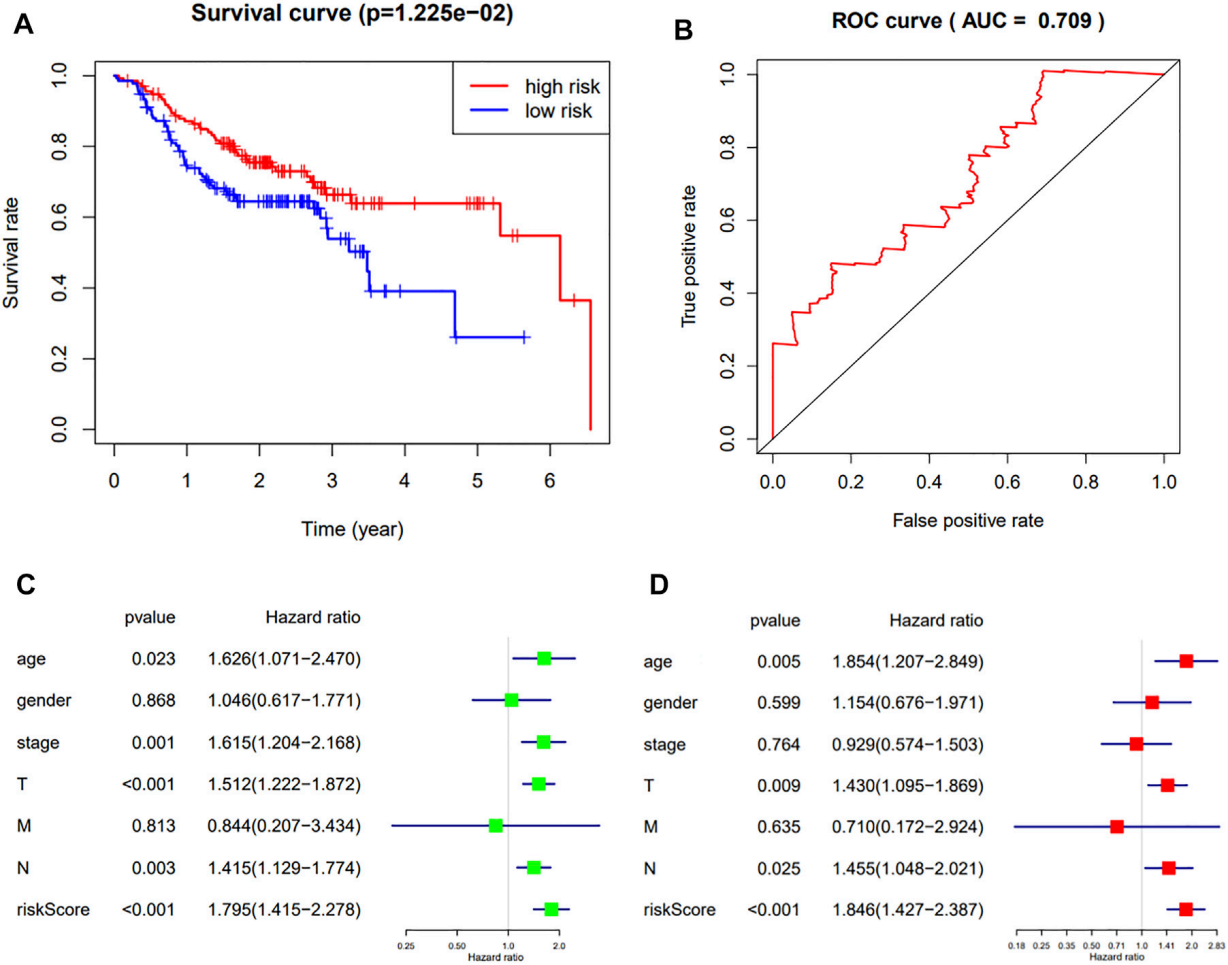
**(A,B)**: The Kaplan-Meier survival curve and ROC curve of HNSCC patients in the external dataset; **(C,D)**: The results of the univariate and multivariate Cox regression analyses in the external dataset.

To further verify the robustness of the 3-gene prognostic model with different data platforms, we used GEO platform data as external datasets, calculating a risk score for each sample in GSE65858, and used the cutoff value circulated in the TCGA cohort to divide the samples into high-risk and low-risk groups. And the survival analysis showed that the high-risk group had a significantly worse overall survival than the low-risk group (*p* = 0.01225, AUC = 0.709) (Figures 6.A,B). The univariate analysis indicated that the age (*p* = 0.023, HR = 1.626, 95%CI = 1.071–2.470), the stage (*p* = 0.001, HR = 1.615, 95% CI = 1.204–2.168), T-staging (*p* < 0.001, HR = 1.512, 95% CI = 1.222–1.872), N-staging (*p* = 0.003, HR = 1.415, 95% CI = 1.129–1.774) and risk score (*p* < 0.001, HR = 1.795, 95% CI = 1.415–2.278) were significantly associated with the OS (Figure 6C).And the multivariate analysis showed that the age (*p* = 0.005, HR = 1.854, 95%CI = 1.207–2.849), T-staging (*p* = 0.009, HR = 1.430, 95%CI = 1.095–1.869), N-staging (*p* = 0.025, HR = 1.455, 95% CI = 1.048–2.021) and risk score (*p* < 0.001, HR = 1.846, 95% CI = 1.427–2.387) were independent prognostic indicators (Figure 6D).

## Discussion

In the past few decades, the 5-years overall survival rate for HNSCC has been worse for the limitations in risk stratification and individual therapeutic strategies selection for HNSCC patients (Chow 2020). The development of HNSCC is a multistep process which involves gradual acquisition of genetic alterations, leading to uncontrolled growth and proliferation of tumor cells. In this study, we performed the differential expression analysis for HNSCC to discover the potential molecular mechanism and signaling pathways for the initiation and progression of HNSCC. Then we establish a three-gene-based prognostic model, which was further validated as an independently predictive factor according to the univariate and multivariate Cox regression analyses.

Three molecular biomarkers, including COL4A1, PLAU and ITGA5 were finally encompassed in our prognostic model. COL4A1 encodes the α1 chain of type IV collagen, which is the most abundant constituents of basement membranes for ECM (Kalluri 2003). The overexpressed COL4A1 could lead to the structural changes of ECM and was significantly associated with the initiation and metastasis of tumors. Deregulated COL4A1 was identified in lots of tumors, such as esophageal squamous cell carcinoma, laryngeal squamous cell carcinoma, hepatocellular carcinoma, and bladder cancer, etc. (Miyake et al., 2017; Chen et al., 2019; Liu et al., 2020; Zhong et al., 2020). Interestingly, HNSCC patients with higher COL4A1 expression showed a better disease-free survival in our study, indicating that COL4A1 could plays an inhibitory role in tumor recurrence. And it has been reported that COL4A1 plays a key role in the recurrence of multiple tumors, such as gastric carcinoma and bladder cancer (Li et al., 2019; Cao et al., 2020). In addition, COL4A1 acted as a protective factor in the prognostic model, further suggesting that the disorder of COL4A1 could contribute to the inhibition effects in the development of HNSCC. But the specific mechanism of COL4A1 in the initiation and recurrence for HNSCC have not been elucidated yet. PLAU encodes for urokinase plasminogen activator (uPA), participating in the activation of plasminogen system (Mahmood et al., 2018). Overexpressed PLAU could increase cell adhesion and migration, which is important in the metastasis and proliferation for tumor cells (Zhao et al., 2020). Besides, the pearson correlation analysis revealed that ITGA5 was highly correlated with PLAU (r = 0.61), indicating that there was a potential mutual promotion between ITGA5 and PLAU. And it is reported that the knockdown of ITGA5 could decrease cell adhesion and promote apoptosis (Murillo et al., 2004).

In the selection of datasets, we chose three array-based datasets as the original data source rather than RNA-seq datasets for some unavoidable reasons. For example, HNSCC samples in RNA-seq datasets are often specially processed (such as specific gene knockout, radiotherapy, and chemotherapy treatment, etc.). And although the RNA-seq datasets could be used to obtain more candidate genes, the sample size of the RNA-seq datasets is always small, which could increase the contingency of our experiment and is not conducive to the applicability of the prognostic model. In addition, the RNA-seq datasets often contains only one sub-category of HNSCC, which does not meet the research focus of this experiment. But it must be admitted that some potential candidates could be lost at the onset of the study for the application of array-based datasets, which might become a limitation of our experiment. Furthermore, we expected that they could be the novel targets for HNSCC patients to improve the prognosis, but their curative effects need to be confirmed by lots of clinical trials in the future.

## Conclusion

In summary, a total of six prognostic genes were identified in this study and a three-gene-based prognostic model for HNSCC patients was successfully established. This prognostic model showed robust efficiency for prognosis prediction and was validated as an independent prognostic factor. It could help clinicians to stratify the risk hazards for HNSCC patients and select a more appropriate treatment plan to improve the prognosis. However, this study is a pure bioinformatics study which only used data in GEO and TCGA databases. Thus, further clinical trials are needed to prove the clinical application value in the future.

## Data Availability

The original contributions presented in the study are included in the article/Supplementary Material, further inquiries can be directed to the corresponding author.
